# Ni_2_Si(P_2_O_7_)_2_

**DOI:** 10.1107/S1600536809021734

**Published:** 2009-06-27

**Authors:** Yahya Toubi, Rachid Essehli, Michal Dušek, Karla Fejfarová, Brahim El Bali

**Affiliations:** a Laboratory of Mineral Solid and Analytical Chemistry, "LCSMA", Department of Chemistry, Faculty of Sciences, University Mohamed I, PO Box 717, 60000 Oujda, Morocco; b Institute of Physics of the ASCR, Na Slovance 2, 182 21 Praha 8, Czech Republic

## Abstract

Dinickel(II) silicon bis­[diphosphate(4−)], Ni_2_Si(P_2_O_7_)_2_, is isotypic with other phosphates of the formula *M*_2_Si(P_2_O_7_)_2_ (*M* =  Co, Cd). All atoms except Si (site symmetry 2) are found in general positions. Ni_2_O_10_ dimers formed from edge-sharing NiO_6_ octa­hedra are linked by corners and O—P—O bridges, forming slabs parallel to (100), which are in turn inter­connected by O—Si—O contacts.

## Related literature

For the structures of isotypic compounds, see: Glaum & Schmidt (1996 ▶) for Co_2_Si(P_2_O_7_)_2_ and Trojan *et al.* (1987 ▶) for Cd_2_Si(P_2_O_7_)_2_. For bond-length and angle data, see: Bostroem (1987 ▶); Durif (1995 ▶). For the extinction correction, see: Becker & Coppens (1974 ▶).

## Experimental

### 

#### Crystal data

Ni_2_Si(P_2_O_7_)_2_
*M* *_r_* = 493.3
Monoclinic, 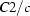

*a* = 16.8615 (9) Å
*b* = 4.8948 (2) Å
*c* = 12.1925 (5) Å
β = 103.693 (4)°
*V* = 977.69 (8) Å^3^
*Z* = 4
Mo *K*α radiation
μ = 4.72 mm^−1^
*T* = 295 K
0.27 × 0.16 × 0.07 mm

#### Data collection

Oxford Diffraction Xcalibur diffractometer with Atlas (Gemini ultra Cu) detector
Absorption correction: analytical (*CrysAlis RED*; Oxford Diffraction, 2006 ▶) *T*_min_ = 0.438, *T*_max_ = 0.829
4978 measured reflections
1013 independent reflections
877 reflections with *I* > 3σ(*I*)
*R*_int_ = 0.023

#### Refinement

*R*[*F* ^2^ > 2σ(*F* ^2^)] = 0.016
*wR*(*F* ^2^) = 0.045
*S* = 1.34
1013 reflections
97 parameters
Δρ_max_ = 0.22 e Å^−3^
Δρ_min_ = −0.23 e Å^−3^

### 

Data collection: *CrysAlis CCD* (Oxford Diffraction, 2006 ▶); cell refinement: *CrysAlis RED* (Oxford Diffraction, 2006 ▶); data reduction: *CrysAlis RED*; program(s) used to solve structure: *Superflip* (Palatinus & Chapuis, 2007 ▶); program(s) used to refine structure: *JANA2006* (Petříček *et al.*, 2006 ▶); molecular graphics: *DIAMOND* (Brandenburg & Putz, 2005 ▶); software used to prepare material for publication: *JANA2006*.

## Supplementary Material

Crystal structure: contains datablocks global, I. DOI: 10.1107/S1600536809021734/mg2073sup1.cif

Structure factors: contains datablocks I. DOI: 10.1107/S1600536809021734/mg2073Isup2.hkl

Additional supplementary materials:  crystallographic information; 3D view; checkCIF report

## Figures and Tables

**Table 1:** Selected bond lengths (Å)

Ni1—O1	1.9631 (18)
Ni1—O4^i^	2.2351 (17)
Ni1—O4^ii^	2.1436 (14)
Ni1—O5^iii^	2.1271 (17)
Ni1—O5^iv^	2.1509 (14)
Ni1—O6	1.9660 (18)
Si1—O2^v^	1.6018 (18)
Si1—O2^vi^	1.6018 (18)
Si1—O7	1.5881 (16)
Si1—O7^vii^	1.5881 (16)
P1—O1	1.4759 (17)
P1—O2	1.564 (2)
P1—O3^viii^	1.5888 (15)
P1—O4	1.5132 (16)
P2—O3	1.5873 (19)
P2—O5	1.5017 (17)
P2—O6	1.4768 (18)
P2—O7	1.5458 (16)

Symmetry codes: (i) 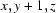; (ii) 
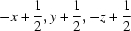
; (iii) 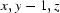; (iv) 
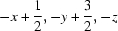
; (v) 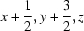; (vi) 
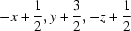
; (vii) 
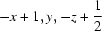
; (viii) 
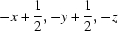
.

**Fig. 1.**
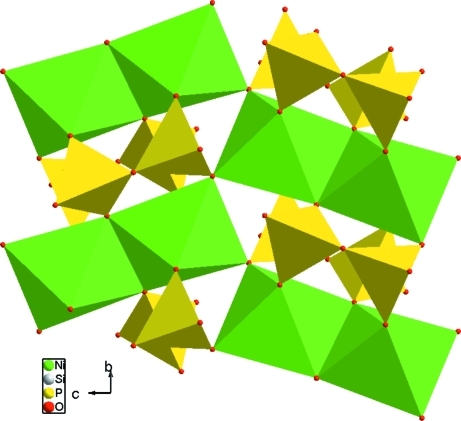
Ni2O10 dimers and their connection in Ni2Si(P2O7)2.

**Fig. 2.**
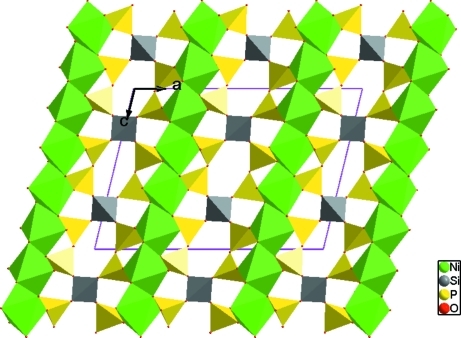
Projection of Ni2Si(P2O7)2 along [010].

**Fig. 3.**
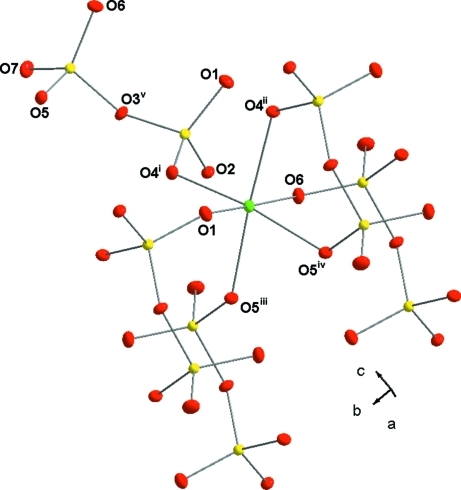
Local coordination geometry around the Ni atom in Ni2Si(P2O7)2. Symmetry codes: (i) x, 1 + y, z, (iii) x, 1 - y, z (ii) -x + 1/2,y + 1/2,-z + 1/2, (iv) -x + 1/2,-y + 3/2,-z, (v) -x + 1/2,-y + 1/2,-z. Displacement ellipsoids are drawn at the 50% probability level.

## supplementary crystallographic information

### Comment

Although the isostructural silicodiphosphates *M*_2_Si(P_2_O_7_)_2_ have been reported for *M* = Fe, Ni, Co, Cu, and Cd, full single-crystal structure determinations have been carried out only for the Co and Cd members (Glaum & Schmidt, 1996; Trojan *et al.*, 1987). The structure of Ni_2_Si(P_2_O_7_)_2_ is reported herein. Its cell parameters are consistent with those obtained earlier from Guinier photographs (Glaum & Schmidt, 1996). The three-dimensional framework is built up of NiO_6_ octahedra, SiO_4_ tetrahedra, and P_2_O_7_ diphosphate groups. Ni_2_O_10_ dimers, formed from pairs of edge-sharing NiO_6_ octahedra, are interconnected through corners (O4) and O–P–O bridges of the diphosphate groups to generate slabs that lie parallel to (100) (Fig. 1). Neighboring slabs are connected through O–Si–O linkages from the SiO_4_ groups (Fig. 2). The NiO_6_ octahedron, whose apices are formed from one bidentate and four monodentate P_2_O_7_ groups (Fig. 3), is strongly distorted, with an average Ni–O distance [2.098 (2) Å] that is between those in Ni_2_P_2_O_7_ (2.114 Å) and Ni_2_SiO_4_ (2.080 Å) (Bostroem, 1987). The P_2_O_7_ group adopts a nearly eclipsed conformation, with an average P–O distance [1.532 (2) Å] that is similar to other diphosphates (Durif, 1995) and a rather small P–O–P angle [132.51 (12) °] owing to its bidentate coordination to the Ni center.

### Experimental

Ni_2_Si(P_2_O_7_)_2_ in the form of powder was prepared by stoichiometric reaction of SiO_2_ (99.9%, Aldrich) and Ni_2_P_4_O_12_ (the latter being obtained from a reaction of NiO and NH_4_H_2_PO_4_ in a 2:4 ratio at 750 °C for 24 h under O_2_ atmosphere). The mixture was heated at 500 °C under Ar atmosphere for 2 days and 700 °C for 36 h with intermediate grindings to ensure complete reaction. Subsequent melting at 1300 °C followed by slow cooling to room temperature at a rate of 5 ° h^-1^ resulted in green crystals of the title compound.

### Crystal data

**Table:**

Ni_2_Si(P_2_O_7_)_2_	*F*(000) = 968
*M**_r_* = 493.3	*D*_x_ = 3.351 Mg m^−^^3^
Monoclinic, *C*2/*c*	Mo *K*α radiation, λ = 0.71073 Å
Hall symbol: -C 2yc	Cell parameters from 3598 reflections
*a* = 16.8615 (9) Å	θ = 2.7–26.5°
*b* = 4.8948 (2) Å	µ = 4.72 mm^−^^1^
*c* = 12.1925 (5) Å	*T* = 295 K
β = 103.693 (4)°	Plate, yellow
*V* = 977.69 (8) Å^3^	0.27 × 0.16 × 0.07 mm
*Z* = 4	

### Data collection

**Table:**

Oxford Diffraction Xcalibur diffractometer with Atlas (Gemini ultra Cu) detector	1013 independent reflections
Radiation source: fine-focus sealed tube	877 reflections with *I* > 3σ(*I*)
graphite	*R*_int_ = 0.023
Detector resolution: 20.7567 pixels mm^-1^	θ_max_ = 26.5°, θ_min_ = 3.4°
Rotation method data acquisition using ω scans	*h* = −20→20
Absorption correction: analytical (*CrysAlis RED*; Oxford Diffraction, 2006)	*k* = −6→6
*T*_min_ = 0.438, *T*_max_ = 0.829	*l* = −15→15
4978 measured reflections	

### Refinement

**Table:**

Refinement on *F*^2^	0 constraints
*R*[*F*^2^ > 2σ(*F*^2^)] = 0.016	Weighting scheme based on measured s.u.'s *w* = 1/[σ^2^(*I*) + 0.0004*I*^2^]
*wR*(*F*^2^) = 0.045	(Δ/σ)_max_ = 0.010
*S* = 1.34	Δρ_max_ = 0.22 e Å^−^^3^
1013 reflections	Δρ_min_ = −0.23 e Å^−^^3^
97 parameters	Extinction correction: B–C type 1 Lorentzian isotropic (Becker & Coppens, 1974)
0 restraints	Extinction coefficient: 370 (80)

### Special details

**Table:**

Experimental. Absorption correction CrysAlisPro; Oxford Diffraction, 2009; Version 1.171.33.34d (release 27-02-2009 CrysAlis171.NET) Absorption correction: analytical, implemented in CrysAlis RED (Oxford Diffraction, 2006).
Refinement. The refinement was carried out against all reflections. The conventional *R*-factor is always based on *F*. The goodness of fit as well as the weighted *R*-factor are based on *F* and *F*^2^ for refinement carried out on *F* and *F*^2^, respectively. The threshold expression is used only for calculating *R*-factors *etc*. and it is not relevant to the choice of reflections for refinement.The program used for refinement, Jana2006, uses the weighting scheme based on the experimental expectations, see _refine_ls_weighting_details, that does not force *S* to be one. Therefore the values of *S* are usually larger than the ones from the *SHELX* program.

### Fractional atomic coordinates and isotropic or equivalent isotropic displacement parameters (Å^2^)

**Table:**

	*x*	*y*	*z*	*U*_iso_*/*U*_eq_	
Ni1	0.247526 (19)	0.35079 (6)	0.12916 (2)	0.00715 (11)	
Si1	0.5	1.16111 (18)	0.25	0.0054 (3)	
P1	0.12621 (4)	−0.13090 (12)	0.13612 (5)	0.00608 (18)	
P2	0.36378 (4)	0.83352 (12)	0.08433 (5)	0.00583 (18)	
O1	0.14379 (10)	0.1598 (3)	0.12002 (13)	0.0100 (5)	
O2	0.04526 (11)	−0.1475 (3)	0.17733 (12)	0.0088 (5)	
O3	0.40484 (11)	0.7811 (3)	−0.01825 (12)	0.0091 (5)	
O4	0.19424 (10)	−0.3018 (3)	0.20675 (12)	0.0088 (5)	
O5	0.29494 (10)	1.0316 (3)	0.04486 (12)	0.0091 (5)	
O6	0.34569 (10)	0.5703 (3)	0.13237 (12)	0.0101 (5)	
O7	0.43615 (10)	0.9697 (3)	0.16829 (13)	0.0116 (5)	

### Atomic displacement parameters (Å^2^)

**Table:**

	*U*^11^	*U*^22^	*U*^33^	*U*^12^	*U*^13^	*U*^23^
Ni1	0.00662 (18)	0.00720 (17)	0.00757 (17)	−0.00117 (13)	0.00154 (12)	0.00048 (11)
Si1	0.0046 (5)	0.0065 (4)	0.0053 (4)	0	0.0012 (3)	0
P1	0.0054 (3)	0.0070 (3)	0.0061 (3)	−0.0008 (2)	0.0019 (2)	−0.0002 (2)
P2	0.0055 (3)	0.0064 (3)	0.0054 (3)	−0.0008 (2)	0.0010 (2)	−0.0003 (2)
O1	0.0076 (9)	0.0082 (8)	0.0146 (8)	−0.0006 (7)	0.0035 (7)	0.0012 (7)
O2	0.0085 (9)	0.0103 (8)	0.0088 (7)	−0.0003 (7)	0.0045 (6)	0.0001 (6)
O3	0.0086 (9)	0.0128 (9)	0.0066 (7)	−0.0004 (7)	0.0030 (6)	−0.0013 (6)
O4	0.0079 (9)	0.0094 (8)	0.0085 (8)	0.0019 (7)	0.0009 (7)	−0.0009 (6)
O5	0.0091 (9)	0.0098 (8)	0.0081 (7)	0.0015 (7)	0.0015 (6)	−0.0008 (7)
O6	0.0090 (9)	0.0099 (8)	0.0112 (8)	−0.0022 (7)	0.0019 (7)	0.0025 (6)
O7	0.0092 (10)	0.0145 (9)	0.0099 (8)	−0.0035 (7)	−0.0002 (7)	−0.0037 (7)

### Geometric parameters (Å, °)

**Table:**

Ni1—O1	1.9631 (18)	Si1—O7^vii^	1.5881 (16)
Ni1—O4^i^	2.2351 (17)	P1—O1	1.4759 (17)
Ni1—O4^ii^	2.1436 (14)	P1—O2	1.564 (2)
Ni1—O5^iii^	2.1271 (17)	P1—O3^viii^	1.5888 (15)
Ni1—O5^iv^	2.1509 (14)	P1—O4	1.5132 (16)
Ni1—O6	1.9660 (18)	P2—O3	1.5873 (19)
Si1—O2^v^	1.6018 (18)	P2—O5	1.5017 (17)
Si1—O2^vi^	1.6018 (18)	P2—O6	1.4768 (18)
Si1—O7	1.5881 (16)	P2—O7	1.5458 (16)
			
O1—Ni1—O4^i^	86.81 (7)	O2^vi^—Si1—O7	110.56 (9)
O1—Ni1—O4^ii^	95.28 (6)	O2^vi^—Si1—O7^vii^	109.83 (8)
O1—Ni1—O5^iii^	93.12 (7)	O7—Si1—O7^vii^	107.67 (10)
O1—Ni1—O5^iv^	89.28 (6)	O1—P1—O2	108.10 (10)
O1—Ni1—O6	174.93 (7)	O1—P1—O3^viii^	111.07 (9)
O4^i^—Ni1—O4^ii^	90.65 (6)	O1—P1—O4	117.37 (9)
O4^i^—Ni1—O5^iii^	176.27 (5)	O2—P1—O3^viii^	98.09 (9)
O4^i^—Ni1—O5^iv^	98.09 (6)	O2—P1—O4	112.97 (9)
O4^i^—Ni1—O6	89.81 (7)	O3^viii^—P1—O4	107.57 (9)
O4^ii^—Ni1—O5^iii^	93.07 (6)	O3—P2—O5	107.46 (9)
O4^ii^—Ni1—O5^iv^	170.37 (7)	O3—P2—O6	109.95 (10)
O4^ii^—Ni1—O6	88.53 (6)	O3—P2—O7	99.69 (9)
O5^iii^—Ni1—O5^iv^	78.19 (6)	O5—P2—O6	118.30 (10)
O5^iii^—Ni1—O6	90	O5—P2—O7	111.28 (9)
O5^iv^—Ni1—O6	87.45 (6)	O6—P2—O7	108.55 (9)
O2^v^—Si1—O2^vi^	108.39 (10)	Si1^ix^—O2—P1	140.73 (11)
O2^v^—Si1—O7	109.83 (8)	P1^viii^—O3—P2	132.51 (12)
O2^v^—Si1—O7^vii^	110.56 (9)	Si1—O7—P2	168.95 (12)

Symmetry codes: (i) *x*, *y*+1, *z*; (ii) −*x*+1/2, *y*+1/2, −*z*+1/2; (iii) *x*, *y*−1, *z*; (iv) −*x*+1/2, −*y*+3/2, −*z*; (v) *x*+1/2, *y*+3/2, *z*; (vi) −*x*+1/2, *y*+3/2, −*z*+1/2; (vii) −*x*+1, *y*, −*z*+1/2; (viii) −*x*+1/2, −*y*+1/2, −*z*; (ix) *x*−1/2, *y*−3/2, *z*.
